# The effect of removing point-of-purchase price discounts on college students’ future tobacco use susceptibility depends on price sensitivity

**DOI:** 10.1093/tbm/ibag055

**Published:** 2026-08-24

**Authors:** Steven C Martino, Claude M Setodji, Michael S Dunbar, Abigail Torbatian, Desmond Jenson, William G Shadel

**Affiliations:** RAND, Pittsburgh, PA, United States; RAND, Pittsburgh, PA, United States; RAND, Pittsburgh, PA, United States; RAND, Pittsburgh, PA, United States; Mitchell Hamline School of Law, Saint Paul, PA, United States; RAND, Pittsburgh, PA, United States

**Keywords:** price sensitivity, tobacco discount information, tobacco advertising, tobacco price promotions, youth tobacco use

## Abstract

**Background:**

Tobacco companies use point-of-sale (POS) discounts and coupons to sustain demand and normalize tobacco use; removing price promotions could counteract these effects.

**Purpose:**

Investigate whether the impact of removing tobacco price discount information from retail POS on young people’s susceptibility to using tobacco products depends on price sensitivity, an individual difference that determines consumer reactions to price levels.

**Methods:**

College students (*N* = 298) participated in an experiment in which they shopped in a replica convenience store that either retained or had removed price discount information from tobacco product advertisements. Before shopping, participants completed questionnaires assessing tobacco use history, susceptibility to cigarette smoking and smokeless tobacco use, and two facets of price sensitivity: price consciousness and sale proneness. Post-shopping susceptibility to smoking cigarettes and using smokeless tobacco were the outcomes.

**Results:**

Previous analyses of these data showed that removing price discount information was associated with lower odds of susceptibility to both cigarette smoking and smokeless tobacco use. In this analysis, we tested whether these effects were moderated by price consciousness and sale proneness. Post-hoc probing of two marginally significant interactions (*P *= .06 and *P *< .08) indicated that removing price discount information was associated with lower odds of cigarette smoking susceptibility only among participants high in sale proneness, and lower odds of smokeless tobacco use susceptibility only among those high in price consciousness.

**Conclusions:**

Removing tobacco price discount information from the POS may reduce the likelihood that young people are susceptible to tobacco use, although this effect may be less evident among those with low price sensitivity.

## Teaser

The results of this study indicate that removing tobacco price discount information from the retail point of sale may make young people less likely to be susceptible to tobacco use, although this effect may be less evident among those with low price sensitivity.

Implications **Practice:** These results highlight the need for complementary strategies to engage youth who are less responsive to price cuts, such as enhanced health communication or restrictions on other forms of promotion.

**Policy:** Findings from this study suggest that removing tobacco price discount information from the retail point of sale may help reduce young people’s susceptibility to tobacco use, particularly among those who are more sensitive to price.

**Research:** This study underscores the importance of considering individual differences when evaluating the effectiveness of tobacco regulatory policies.

## Introduction

Tobacco use remains the leading cause of preventable death in the United States, claiming an estimated 490 000 lives each year [1]. Because nearly all tobacco use begins during adolescence and young adulthood [2], youth and young adults are a primary focus of tobacco prevention and control efforts. Despite considerable progress [3], underage tobacco use remains high: in 2023, more than 13% of youth aged 12–20 used at least one tobacco product in the past month [4].

Raising tobacco prices through taxation reduces initiation and encourages cessation among young people [5, 6]. In the United States, tobacco products are taxed at both federal and state levels. As of 2024, the federal cigarette tax was $1.01 per pack and the average state tax was $1.93 [7]. Although federal taxes on other tobacco products are comparably lower, state taxes can be considerable. For example, California, Colorado, Maine, Utah, and Vermont have taxes on other tobacco products that are equivalent to their tax on cigarettes [7].

To offset taxes and sustain demand, tobacco companies spend billions of dollars annually on point-of-sale (POS) coupons, discounts, and related incentives [5, 8–10]. Consumers are therefore routinely exposed to signs advertising discounted prices and special offers [11]. This promotional activity increases demand and may normalize tobacco use among young people even when it does not result in an immediate purchase [12, 13].

Theories of consumer behavior suggest that the effects of price promotion strategies by tobacco companies—and the effects of policies designed to limit the use of such strategies—may depend on consumer price sensitivity. Price sensitivity refers to the degree to which individual consumers react, in terms of attention and purchasing behavior, to price levels and changes in price levels [14]. Lichtenstein and colleagues conceptualized price sensitivity as a multifaceted construct encompassing seven dimensions of consumer response to pricing information and promotions [15–17]. For example, they defined value consciousness as a concern with the ratio of quality received to price paid in a consumer transaction, price consciousness as a general focus on paying low prices, sale proneness as an increased propensity to exhibit interest in a product purely because it is on sale, and prestige sensitivity as a concern about what a purchase signals to other people about the purchaser. Lichtenstein and colleagues developed multi-item scales to assess each of these facets of price sensitivity and demonstrated their internal consistency and discriminant and predictive validity with respect to consumer purchasing behavior [17].

Individuals high in price sensitivity tend to monitor price fluctuations, seek deals, and adjust purchasing behavior accordingly, e.g. by switching brands, delaying purchases, or refraining from buying when prices increase [14, 18]. Accordingly, tobacco price promotions at the POS may disproportionately attract the attention of highly price-sensitive consumers, whereas those low in price sensitivity may be less influenced by such cues. Although prior studies have examined demographic characteristics and levels of tobacco use (as a proxy for nicotine dependence) as moderators of responsiveness to tobacco price changes [19–22], no study has yet explored whether price sensitivity, as a stable psychological trait, moderates responses to the tobacco industry’s price promotion strategies or regulatory policies designed to counteract those strategies. Such work can clarify population-level policy effects and identify subgroups who may require complementary interventions [23, 24].

### Purpose of the current study

The current study addressed this gap using data from an experimental investigation of the effects of removing tobacco product price discount information from a simulated convenience store on college students’ future tobacco use susceptibility. Prior analyses of these data showed that removing tobacco price discount information from the store lowered the odds of being susceptible to both cigarette smoking and smokeless tobacco use [25, 26]. Here, we examined whether price sensitivity moderates those effects. We hypothesized that participants with higher price sensitivity would be more responsive to the removal of price discount information than those with lower price sensitivity.

## Methods

### Participants

A multimedia (e.g. bus ads, paper flyers, radio, and social media) advertising campaign was used to recruit college students in the Pittsburgh, PA area from October 2022 to February 2024. Advertising indicated that the study pertained to college students’ convenience store shopping habits; advertisements did not mention smoking or other forms of tobacco use. Interested individuals completed an online screener that assessed eligibility criteria, which included being 18–20 years old (i.e. below the minimum age required to purchase tobacco products); enrolled in a 2- or 4-year college or professional training program; and not having previously participated in a RAND StoreLab study (see below). The sample size for this study was determined by the parent randomized experiment from which these secondary analyses were drawn [25, 26].

During the informed consent process, eligible individuals were told that the study concerned convenience store shopping habits and that some study details could not be disclosed until after their participation because doing so could jeopardize study results. Participants’ written consent indicated their agreement to participate without knowledge of some study details.

A total of 298 eligible college students were randomized to an experimental condition (148 to the price discount information absent condition and 150 to the price discount information present condition), completed the study, and were debriefed. An additional 106 students were deemed eligible and consented to participate but failed to show up for the in-person session. There were no statistically significant differences between study completers and non-completers on age (*P *= .23), gender (*P *= .06), race (*P *= .52), ethnicity (*P *= .87), or socioeconomic status (*P *= .34), as measured by the four-item Family Affluence Scale, a reliable and valid measure of socioeconomic status in young people [27, 28].

### Experimental setting

This study took place in the RAND StoreLab (RSL), which is a replica of a mid-sized convenience store that occupies 1500 square feet inside a Pittsburgh, PA office building. The store, which stocks over 650 different products (e.g. dairy, snack foods, beverages, and tobacco), is accessible only to study participants. Posters are displayed throughout the store, advertising various products available in the store. Product displays and stickers convey prices that are consistent with local norms.

For this study, a 58.5-square-foot tobacco power wall was situated behind the cashier counter. This display included 120 cigarette pack-facings for brands and sub-brands that command a large segment of the US marketplace (e.g. Marlboro, Newport, Camel, and Winston), 24 smokeless tobacco/nicotine pouch facings (including Zyn, Skoal, and other popular brands), 12 package facings for little cigars/cigarillos (e.g. Black & Mild), and 32 electronic nicotine delivery system (ENDS) pack facings (e.g. Juul, Blu Menthol). Prices and small posters for various available products were also displayed on the power wall.

### Experimental conditions

The two experimental conditions differed in whether price-discount information was present or absent on posters displayed on the outside-facing doors of the store. In both conditions, these doors displayed 16 posters advertising tobacco products: eight for cigarettes, four for ENDS products, two for little cigars/cigarillos, and two for smokeless tobacco products. In the *discounts present* condition, exactly half of posters within a product type contained price-discount information; in the *discounts absent* condition, no price-discount information was displayed. Posters for each condition were presented in four different configurations, randomly chosen, to minimize any positioning effects. As positioning was not related to the dependent measures, we do not discuss it further here.

### Procedures

Before attending an in-person shopping session, participants completed an online baseline questionnaire that assessed demographics; tobacco product use, cognitions, and susceptibility; and price sensitivity. The baseline questionnaire also included a number of filler items to disguise the purpose of the study, i.e. items about soda, potato chips, and other store products that mimicked the structure of the items pertaining to tobacco use, and questions about convenience store shopping habits.

Upon arrival, participants received a $20 gift card and were told to shop as they normally would, select at least one item, and check out as in any convenience store. Instructions regarding the shopping task were delivered to participants outside the store as they viewed the doors that displayed the experimental stimulus. These instructions took about 30 s to deliver, the average amount of time our pretesting indicated that the doors of convenience stores are visible to customers as they approach from the street or a parking lot.

Each participant shopped in the store alone. A research assistant who was not involved in the consent or check-in process acted as the cashier. When the participant was finished shopping, the cashier scanned the selected items, placed them in a bag, and scanned the study-issued gift card. No participant attempted to purchase a tobacco product during the study; if any participant had attempted to purchase a tobacco product, the cashier would have requested verification of their age and denied the attempt (all participants were under the legal age for making such a purchase).

### Dependent measures

Cigarette smoking susceptibility and smokeless tobacco use susceptibility were each assessed using a well-validated and widely used three-item measure [29–31]. Participants were asked, “Do you think you will try [a cigarette/smokeless tobacco] anytime soon?”; “Do you think you will try [a cigarette/smokeless tobacco] anytime in the next year?”; and “If one of your best friends offered you [a cigarette/smokeless tobacco], would you [smoke/use] it?” Responses to each item were made on a 1 (*definitely not*) to 10 (*Definitely yes*) scale and summed. Our use of a 1–10 response scale for these adapted susceptibility items is consistent with recent psychometric work evaluating tobacco susceptibility measurement in contemporary retail contexts [32]. Alpha reliability for each three-item scale using continuously scored items was ≥0.89. Because the distribution of scores on each summary index was highly skewed (most participants had the lowest possible score: 3), we dichotomized each scale for analysis. We re-coded scores of 3 (the lowest possible score) to 0 (no susceptibility) and scores >3 to 1 (any susceptibility), a common scoring convention for these items [32]. Susceptibility scores derived from these items prospectively predict cigarette smoking [30] and smokeless tobacco use [33]. E-cigarette and little cigar use susceptibility were also assessed in the parent experiment; because the manipulation did not significantly affect either outcome [26], this secondary analysis focused on the two outcomes that did show significant effects.

### Hypothesized moderator

We assessed price consciousness and sale proneness, two facets of price sensitivity that were most relevant to our study manipulation, using a set of items developed and validated by Lichtenstein et al. [17]. The full scales include five price consciousness items and six sale proneness items We reduced each scale to three items by removing items with wording similar to retained items while preserving coverage of the constructs of price consciousness and sale proneness (see Table 2). We also made minor wording changes to improve consistency across items and align them with the convenience store context. These modifications were intended to minimize response burden and reduce the likelihood that participants would infer the specific study hypotheses from an extensive set of price- and sale-focused questions administered before the shopping task. Respondents answered these six items using a 1 (*strongly disagree*) to 10 (*strongly agree*) scale. We selected these two scales from among the seven that Lichtenstein et al. [17] developed because they were most relevant to our manipulation of price incentive information on store posters.

### Control variables

Because demographic characteristics were balanced across conditions and did not predict either outcome in prior analyses [25, 26], we included only two control variables in the regression analyses described below: any lifetime tobacco use and participants’ baseline standing on the relevant dependent measure. Lifetime tobacco use was assessed with four questions: “Have you ever smoked/used [cigarettes/smokeless tobacco/little cigars or cigarillos/ENDS] in your life? [34].” Example brands were given for each product type; response options were “no” or “yes.” Lifetime tobacco use was coded “0” for participants who indicated that they had never used any of these products; it was coded “1” for participants who indicated that they had used one or more products in their lifetime.

### Analysis

We began by conducting an exploratory factor analysis (EFA) with maximum likelihood extraction and Geomin (oblique) factor rotation [35] to investigate the dimensionality of the six continuously scored (1–10) price sensitivity items. A scree plot and goodness-of-fit indices were used to determine the number of factors. Model fit was evaluated using standards proposed by Hu and Bentler [36] and MacCallum et al. [37]: comparative fit index (CFI) ≥ 0.95, root mean square error of approximation (RMSEA) ≤ 0.08, and standard root mean square residual (SRMR) ≤ 0.08. Next, we estimated logistic regression models predicting each outcome from experimental condition (0 = price discounts present, 1 = price discounts absent), price sensitivity, and the covariates listed above. These models included an interaction between experimental condition and price sensitivity to evaluate our hypothesis about moderation. Analyses used complete cases; depending on the outcome, 8–10 observations were omitted due to missing data. Analyses were performed using SAS version 9.4 [38].

## Results

### Sample characteristics

Participants were an average age of 19.1 years (SD = 0.78). Slightly more than two thirds (70.0%) were female. About half (49.7%) were White, 34.5% were Asian or Pacific Islander, 7.7% were Black, 6.1% were Multiracial, and 1.7% were some other race. Approximately 7.8% of participants identified as Hispanic or Latino. The vast majority (87.6%) of participants indicated that at least one of their parents had graduated college. About half the sample (50.7%) stated that they had never used a tobacco product. Nearly a third (28.6%) indicated that they had used a tobacco product in their lifetime but were not a current user of any tobacco product. The remainder (20.7%) were current users of one or more tobacco products. At baseline (pre-shopping), 43.6% of participants indicated some level of susceptibility to cigarette smoking and 25.0% indicated some level of susceptibility to smokeless tobacco use.

### Factor analysis of price sensitivity items

**Figure 1:**
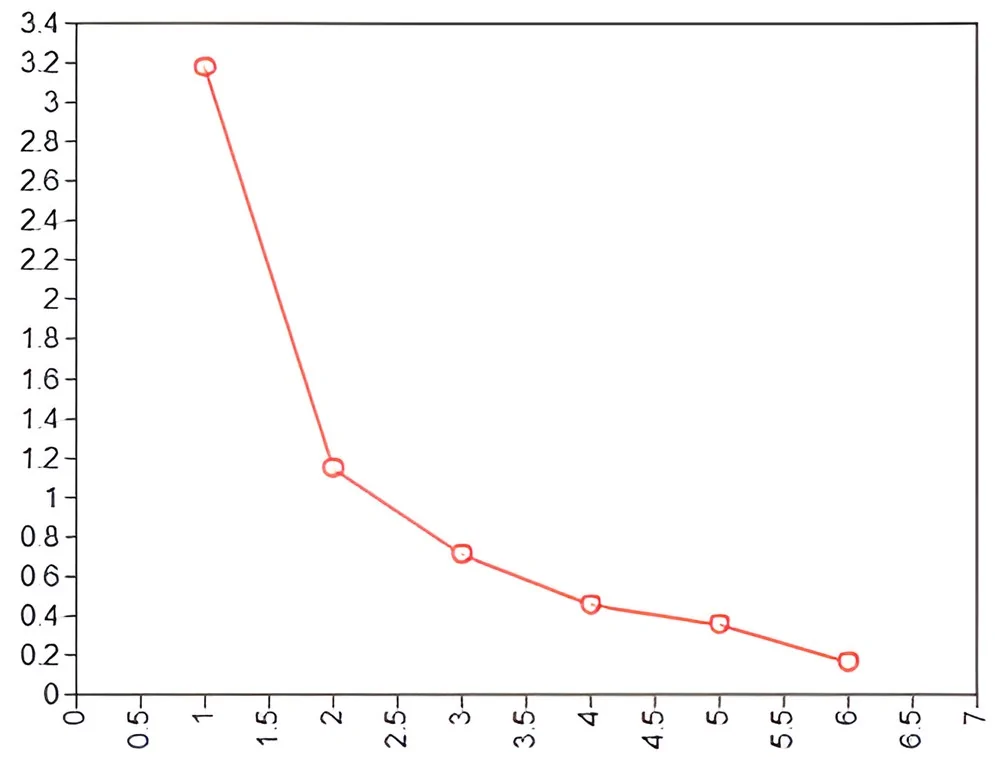
Scree plot: Exploratory factor analysis of shortened price sensitivity scale.

The EFA of the six price sensitivity items suggested that two factors were needed to explain the covariances observed among the items. The scree plot (Fig. 1) supported retaining two factors, which explained 97.2% of total variance. Goodness of fit indices revealed that the two-factor solution fit the data well, CFI = 0.99, RMSEA = 0.08, SRMR = 0.02, and did so considerably better than the one-factor solution, CFI = 0.85, RMSEA = 0.38, SRMR = 0.13. Table 1 shows the loadings for each of the six price sensitivity items on these two factors. Using a cut point of |0.40| [39], the three price consciousness items loaded on the first factor, while two of the three sale proneness items loaded on the second. One of the sale proneness items did not load strongly on either factor. We nevertheless retained this item in the sale proneness factor score because it was conceptually consistent with that construct; the corresponding three-item set showed adequate internal consistency (α = 0.75). The three price consciousness items also showed adequate internal consistency (α = 0.79). Based on this model, we extracted two factor scores for all participants, one representing price consciousness and the other sale proneness.

**Table 1:** Pattern factor loadings for a two-factor model of items in the shortened price sensitivity scale.

Hypothesized domain	Item	Factor 1	Factor 2
Price consciousness			
	I am willing to go to extra effort to find low prices.	**0.65**	0.19
	I will shop at more than one convenience store to take advantage of low prices.	**0.72**	−0.05
	The time it takes to find low prices is usually worth the effort.	**0.85**	0.00
Sale proneness			
	If a product is on sale, that can be a reason for me to buy it.	0.34	0.28
	I have favorite brands, but most of the time I buy the brand that’s on sale.	0.07	**0.80**
	I am more likely to buy brands that are on sale.	−0.01	**0.99**

Items were answered using a 1 (*strongly disagree*) to 10 (*strongly agree*) scale. Maximum likelihood extraction and Geomin rotation were used. Loadings >|0.40| are bolded. Overall fit statistics for the model were: CFI = 0.99, RMSEA = 0.08, SRMR = 0.02. The inter-factor correlation was 0.43.

For ease of exposition, we dichotomized each of these factor scores at the first (lowest) quartile to create low and high price consciousness/sale proneness groups. We selected the first quartile because young people tend to be highly price sensitive, including with regard to tobacco pricing [19, 22, 40, 41]. Young adults also tend to have less disposable income and lower levels of nicotine dependence (if they are tobacco users) than older groups [42, 43], two factors known to increase tobacco price sensitivity [44–48]. Cutting the distribution at the first quartile ensured that the low price-sensitivity group was low on this dimension in absolute terms.

### Regression analyses

Table 2 shows the results of our logistic regression models predicting post-shopping cigarette smoking and smokeless tobacco use susceptibility. We estimated separate regression models to evaluate whether price consciousness or sale proneness moderated the previously observed effect of removing tobacco product price discount information from the store on the odds of being susceptible to using these products. Because these models include an interaction, estimated log odds are reported instead of odds ratios for inference. As can be seen in Table 2, there was a marginally significant interaction between assignment to the price discount information absent condition and high price consciousness on smokeless tobacco use susceptibility. There was also a marginally significant interaction between assignment to the price discount information absent condition and high sale proneness on cigarette smoking susceptibility.

**Table 2:** Logistic regression models predicting post-shopping cigarette smoking and smokeless tobacco use susceptibility from experimental condition and two facets of price sensitivity.

Predictor	Cigarette smoking susceptibility (*n* = 290)			Smokeless tobacco use susceptibility (*n* = 288)		
	Log odds	95% CI	*P*	Log odds	95% CI	*P*
Experimental condition: Price discount information absent^a^	−0.21	−1.59, 1.17	.76	0.33	−1.11, 1.77	.65
High price consciousness^b^	0.40	−0.79, 1.59	.51	0.33	−0.92, 1.59	.60
Price discount information absent x high price consciousness	−0.70	−2.27, 0.86	.38	−1.49	−3.18, 0.2	.08
Cigarette smoking susceptibility, pre-shopping	3.32	2.62, 4.03	.00	3.10	2.35, 3.84	.00
Any lifetime tobacco use	0.89	0.21, 1.57	.01	0.59	−0.14, 1.32	.11
Experimental condition: Price discount information absent^a^	0.48	−0.95, 1.9	.51	0.09	−1.39, 1.57	.91
High sale proneness^b^	1.18	−0.04, 2.4	.06	0.13	−1.08, 1.34	.83
Price discount information absent x high sale proneness	−1.61	−3.28, 0.06	.06	−1.09	−2.83, 0.64	.22
Smokeless tobacco use susceptibility, pre-shopping	3.47	2.73, 4.22	.00	3.14	2.37, 3.91	.00
Any lifetime tobacco use	0.76	0.07, 1.45	.03	0.52	−0.22, 1.25	.17

Because these models include an interaction, log odds estimates are reported and used for inference.

Abbreviation: CI, confidence interval.

a Versus price discount information present.

b Above the first quartile of the factor score distribution.

To interpret these marginally significant interactions, we estimated logistic regression models stratified by the relevant moderator. We report odds ratios for these models, which contained the same control variables as the unstratified models. Stratified models are a common post hoc approach for probing interactions by clarifying the association between the focal predictor and the outcome at different levels of the moderator [49].

Table 3 shows that, among participants high in sale proneness, removing discount information was associated with lower odds of cigarette smoking susceptibility. In contrast, among the group with low sale proneness, there was no evidence that the presence or absence of price discounts affected the likelihood of being susceptible to cigarette smoking. Likewise, Table 4 shows that, among the group with high price consciousness, removing product discount information was associated with lower odds of susceptibility to smokeless tobacco use. In contrast, among the group with low price consciousness, there was no evidence that the presence or absence of price discounts affected the likelihood of being susceptible to smokeless tobacco use.

**Table 3:** Logistic regression model results predicting post-shopping cigarette smoking susceptibility from experimental condition, stratified by sale proneness.

Predictor	High sale proneness^a^ (*n* = 217)			Low sale proneness^b^ (*n* = 73)		
	OR	95% CI	*P*	OR	95% CI	*P*
Experimental condition: Price discount information absent^c^	0.32	0.14, 0.76	.01	2.57	0.58, 11.39	.22
Cigarette smoking susceptibility, pre-shopping	31.00	13.05, 73.64	<.001	62.35	10.30, 377.32	<.001
Any lifetime tobacco use	3.10	1.42, 6.74	.004	0.46	0.08, 2.72	.39

a Above the first quartile of the factor score distribution.

b At or below the first quartile on the factor score distribution.

c Compared with price discount information present.

**Table 4:** Logistic regression model results predicting post-shopping smokeless tobacco use susceptibility from experimental condition, stratified by price consciousness.

Predictor	High price consciousness^a^ (*n* = 217)			Low price consciousness^b^ (*n* = 71)		
	OR	95% CI	*P*	OR	95% CI	*P*
Experimental condition: Price discount information absent^c^	0.33	0.14, 0.77	.01	1.49	0.30, 7.31	.63
Smokeless tobacco use susceptibility, pre-shopping	19.00	8.15, 44.29	<.001	39.20	7.65, 200.97	<.001
Any lifetime tobacco use	1.69	0.73, 3.90	.22	2.50	0.52, 12.04	.25

a Above the first quartile of the factor score distribution.

b At or below the first quartile on the factor score distribution.

c Compared with price discount information present.

## Discussion

In this study, we examined whether individual differences in price sensitivity—specifically price consciousness and sale proneness—moderated previously established effects of removing tobacco price discount information from the retail POS on college students’ susceptibility to cigarette and smokeless tobacco use [25, 26]. The findings suggest that these effects may vary by price sensitivity.

Specifically, post hoc probing of marginally significant interactions suggested that removing tobacco price discount information was associated with lower odds of cigarette smoking susceptibility among youth with higher levels of sale proneness but not among youth with lower levels of sale proneness. Similarly, removal of tobacco price discount information was associated with lower odds of susceptibility to smokeless tobacco use among youth with higher levels of price consciousness but not among youth with lower levels of price consciousness. Because these interaction effects were only marginally significant, these subgroup patterns should be interpreted cautiously.

Even so, this analysis extends prior reports from this experiment [25, 26] by examining whether the previously reported main effects differ across individuals. Identifying such heterogeneity can clarify the boundaries of policy effectiveness and indicate when complementary strategies may be needed. If replicated, these findings would suggest that policies eliminating tobacco price promotions may be more effective for youth who are especially responsive to price cues and less effective for those who are not. For individuals low in price sensitivity, other regulatory strategies may be needed to reduce susceptibility, such as limiting broader tobacco advertising exposure or increasing attention to health risks through graphic warning labels.

The distinct moderation patterns observed for the two tobacco products may reflect differences in both the nature of the price sensitivity constructs and the products’ price structures. Cigarettes are relatively expensive compared to other tobacco products and typical convenience store items. As a result, cigarette discounts may be particularly salient to individuals high in sale proneness, whose interest is drawn to products when they are on sale. In contrast, highly price-conscious consumers may remain deterred by the relatively high absolute cost of cigarettes, even when discounts are present. Smokeless tobacco products, by comparison, are typically less expensive, which may make them more appealing to consumers who prioritize affordability. Under that interpretation, removing discount information may be especially relevant to price-conscious individuals considering these products. These explanations are speculative, and future research should examine how specific facets of price sensitivity interact with product price and promotion strategies.

Several limitations should be considered when interpreting these findings. First, this study is a secondary analysis of a previously published randomized experiment. Although this allowed us to answer a novel moderation question, the current analysis should be understood in that context. Second, the key interaction effects were marginally significant; accordingly, the findings should be viewed as suggestive rather than conclusive. Third, although tobacco use susceptibility is a valid predictor of future behavior [33], actual use was not measured. In addition, assessing tobacco susceptibility before the shopping task may have increased the salience of tobacco products for some participants, although filler items were used to reduce this possibility and any such effect would be expected to operate similarly across conditions. Fourth, although the RSL closely replicated a typical convenience store in Western Pennsylvania, it remains a controlled environment, and findings may not fully reflect the effects of removing tobacco price discount information in real-world settings. Fifth, about a quarter of consented participants did not complete study procedures. Although completers and non-completers did not differ on available demographic characteristics, they may have differed on unmeasured factors relevant to tobacco susceptibility or price sensitivity, which could have introduced selection bias. Finally, our sample was recruited from a single geographic area and consisted of college students below the legal age for purchasing tobacco products. College students are an ideal population for evaluating policies that prohibit display of price promotion information at retail POS because they often shop at convenience stores [50], are key targets of POS tobacco marketing [51], and are at an age when regular tobacco use is typically established [2]. However, they also tend to be highly price sensitive overall, which complicates tests of variation in price sensitivity. Our having to dichotomize the sample at the first quartile of the response distribution for each aspect of price sensitivity to create groups that were meaningfully low in price sensitivity resulted in relatively small subgroup sample sizes.

## Conclusions

Despite these limitations, this study contributes to the tobacco control literature by providing initial evidence that price sensitivity may shape young people’s responses to removal of price discount information at the POS. If confirmed, these findings would suggest that complementary strategies may be needed for youth who are less sensitive to price cues, potentially including stronger health communication, restrictions on other forms of promotion, or educational interventions emphasizing tobacco’s harms.

## Funding sources

This study was funded by the National Cancer Institute of the National Institutes of Health (NIH) under Award Number R01CA236608. Given that this manuscript is the result of funding by the NIH, it is subject to the NIH Public Access Policy. Through acceptance of this federal funding, NIH has been given a right to make this manuscript publicly available in PubMed Central upon the Official Date of Publication, as defined by NIH.

## Role of the funder

The funders had no role in the design and conduct of the study; collection, management, analysis, and interpretation of the data; preparation, review, or approval of the manuscript; or decision to submit the manuscript for publication.

## Data Availability

De-identified data from this study are not available in a public archive but could be made available (as allowable per institutional IRB standards) via reasonable request to the corresponding author.

## Welfare of animals

The study reported in this article did not involve animals.

## References

[1] US Department of Health and Human Services (USDHHS). Eliminating Tobacco-Related Disease and Death: Addressing Disparities—A Report of the Surgeon General. Atlanta, GA: USDHHS, Centers for Disease Control and Prevention, National Center for Chronic Disease Prevention and Health Promotion, Office of Smoking and Health, 2024.

[2] National Center for Chronic Disease Prevention and Health Promotion, Office of Smoking and Health. Preventing Tobacco Use Among Youth and Young Adults: A Report of the Surgeon General. Atlanta, GA: Centers for Disease Control and Prevention, 2012. 22876391

[3] Health, United States: Tobacco Use. Hyattsville, Maryland, U.S.: National Center for Health Statistics, 2024. https://www.cdc.gov/nchs/hus/topics/tobacco-use.htm

[4] Substance A , Mental H, Services A. Key Substance Use and Mental Health Indicators in the United States: Results from the 2023 National Survey on Drug Use and Health (HHS Publication No. PEP24-07-021). Rockville, MD: Center for Behavioral Health Statistics and Quality, SAMHSA, 2024.

[5] Chaloupka FJ , Cummings KM, Morley CP, Horan JK. Tax, price and cigarette smoking: evidence from the tobacco documents and implications for tobacco company strategies. Tob Control 2002;11:I62–72. 10.1136/tc.11.suppl_1.i62 11893816 PMC1766067

[6] Sharbaugh MS , Althouse AD, Thoma FW et al Impact of cigarette taxes on smoking prevalence from 2001-2015: a report using the behavioral and risk factor surveillance survey (BRFSS). PLoS One 2018;13:e0204416. 10.1371/journal.pone.0204416 30235354 PMC6147505

[7] *Cigarette and Tobacco Taxes*. American Lung Association, 2024. https://www.lung.org/policy-advocacy/tobacco/tobacco-taxes (19 February 2026, date last accessed).

[8] Loomis BR , Farrelly MC, Mann NH. The association of retail promotions for cigarettes with the master settlement agreement, tobacco control programmes and cigarette excise taxes. Tob Control 2006;15:458–63. 10.1136/tc.2006.016378 17130375 PMC2563678

[9] US Federal Trade Commission. Federal Trade Commission Cigarette Report for 2022. Washington, DC: US Federal Trade Commission, 2023.

[10] US Federal Trade Commission. Federal Trade Commission Smokeless Tobacco Report for 2022. Washington, DC: US Federal Trade Commission, 2023.

[11] *Pricing Policies for Tobacco Products: Death on a Discount*. Public Health Law Center at Mitchell Hamline Law School, 2024. https://www.publichealthlawcenter.org/sites/default/files/resources/Death-on-a-Discount.pdf (19 February 2026, date last accessed).

[12] DeCicca P , Kenkel D, Mathios A. Putting out fires: will higher taxes reduce the onset of youth smoking? J Political Econ 2002;110:144–69. 10.1086/324386

[13] Slater SJ , Chaloupka FJ, Wakefield M et al The impact of retail cigarette marketing practices on youth smoking uptake. Arch Pediatr Adolesc Med 2007;161:440–5. 10.1001/archpedi.161.5.440 17485618

[14] Goldsmith RE , Newell SJ. Innovativeness and price sensitivity: managerial, theoretical, and methodological issues. J Prod Brand Manag 1997;6:163–74. 10.1108/10610429710175682

[15] Lichtenstein DR , Bloch P, Black WC. Correlates of price acceptability. J Consum Res 1988;15:243–52. 10.1086/209161

[16] Lichtenstein DR , Netemeyer RG, Burton S. Distinguishing coupon proneness from value consciousness: an acquisition-transaction utility theory perspective. J Mark 1990;54:54–67. 10.1177/00222429900540030

[17] Lichtenstein DR , Ridgway NM, Netemeyer RG. Price perceptions and consumer shopping behavior: a field study. J Mark Res 1993;30:234–45. 10.2307/3172830

[18] Han S , Gupta S, Lehmann DR. Consumer price sensitivity and price thresholds. J Retail 2001;7:435456. 10.1016/S0022-4359(01)00057-4

[19] Chaloupka FJ , Wechsler H. Price, tobacco control policies and smoking among young adults. J Health Econ 1997;16:359–73. 10.1016/S0167-6296(96)00530-9 10169306

[20] Chen C-M , Chang K-L, Lin L. Re-examining price sensitivity of demand for cigarettes with quantile regression. Addict Behav 2013;38:2801–4. 10.1016/j.addbeh.2013.07.003 24018220

[21] Kyung Y , Farooqui MA. Differential subjective responsiveness to a future cigarette price increase among South Korean youth smokers. Nicotine Tob Res 2012;14:209–16. 10.1093/ntr/ntr187 22025542

[22] Siahpush M , Heller G, Singh G. Lower level of occupation, income, education are strongly associated with a longer smoking duration: multivariate results from the 2001 Australian National Drug Strategy survey. Public Health 2005;119:1105–10. 10.1016/j.puhe.2005.03.004 16085150

[23] Kowitt SD , Goldstein AO, Schmidt AM et al Attitudes toward FDA regulation of newly deemed tobacco products. Tob Regul Sci 2017;3:504–15. 10.18001/TRS.3.4.10 29450218 PMC5810966

[24] Rose SW , Emery SL, Ennett S et al Public support for family smoking prevention and tobacco control act point-of-sale provisions: results of a national study. Am J Public Health 2015;105:e60–7. 10.2105/AJPH.2015.302751 PMC4566565 26270303

[25] Dunbar MS , Setodji CM, Martino SC et al Removing tobacco price discounts from the retail point-of-sale exterior reduces young adults’ future cigarette smoking susceptibility: an experimental investigation. Addict Behav 2026;177:108653. 41759302 10.1016/j.addbeh.2026.108653 PMC13044973

[26] Shadel WG , Setodji CM, Martino SC et al Removing price discounts from the tobacco retail environment: effects on college students’ risk of using smokeless tobacco, little cigars, and electronic delivery devices. J Stud Alcohol Drugs 2026;87:231–40. 40637109 10.15288/jsad.24-00432 PMC12288586

[27] Boyce W , Torsheim T, Currie C et al The family affluence scale as a measure of national wealth: validation of an adolescent self-report measure. Soc Indic Res 2006;78:473–87. 10.1007/s11205-005-1607-6

[28] Martin-Storey A , Marcellin S, Purtell KM et al “It’s about having money, but also happiness:” a qualitative investigation of how adolescents understand subjective status in themselves and others. J Adolesc 2018;68:198–206. 10.1016/j.adolescence.2018.08.004 30118950

[29] Choi WS , Gilpin EA, Farkas AJ et al Determining the probability of future smoking among adolescents. Addiction 2001;96:313–23. 10.1046/j.1360-0443.2001.96231315.x 11182877

[30] Pierce JP , Choi WS, Gilpin EA et al Validation of susceptibility as a predictor of which adolescents take up smoking in the United States. Health Psychol 1996;15:355–61. 10.1037//0278-6133.15.5.355 8891714

[31] Shadel WG , Martino SC, Setodji CM et al Hiding the tobacco power wall reduces cigarette smoking risk in adolescents: using an experimental convenience store to assess tobacco regulatory options at retail point-of-sale. Tob Control 2015;25:679–84. 10.1136/tobaccocontrol-2015-052529 PMC4877296 26598502

[32] Setodji CM , Martino SC, Dunbar MS et al Measuring susceptibility to use tobacco in an increasingly complex consumer marketplace: how many questions do we really need? Psychol Addict Behav 2025;39:127–38. 10.1037/adb0000997 38421778 PMC11358647

[33] Vigorita MW , Smith A, Farrelly M et al Predictive validity of the original and expanded susceptibility scales for smokeless tobacco. Addict Behav 2022;130:107286. 10.1016/j.addbeh.2022.107286 35231844 PMC13014888

[34] Hyland A , Ambrose BK, Conway KP et al Design and methods of the population assessment of tobacco and health (PATH) study. Tob Control 2017;26:371–8. 10.1136/tobaccocontrol-2016-052934 27507901 PMC5299069

[35] Browne MW. An overview of analytic rotation in exploratory factor analysis. Multivar Behav Res 2001;36:111–50. 10.1207/S15327906MBR3601_05

[36] Hu L , Bentler PM. Cutoff criteria for fit indexes in covariance structure analysis: conventional criteria versus new alternatives. Struct Equ Model 1999;6:1–55. 10.1080/10705519909540118

[37] MacCallum RC , Browne MW, Sugawara HM. Power analysis and determination of sample size for covariance structure modeling. Psychol Methods 1996;1:130–49. 10.1037/1082-989X.1.2.130

[38] SAS Institute, Inc. SAS/STAT® 9.4: User’s Guide. Cary, NC: SAS Institute Inc., 2004.

[39] Tabachnick BG , Fidell LS. Using Multivariate Statistics, 5th edn. Boston, MA: Allyn & Bacon, 2007.

[40] Kostova D , Ross H, Blecher E et al Is youth smoking cessation responsive to cigarette prices evidence from low- and middle-income countries. Tob Control 2011;20:419–24. 10.1136/tc.2010.038786 21737858

[41] Zhang B , Cohen J, Ferrence R et al The impact of tobacco tax cuts on smoking initiation among Canadian young adults. Am J Prev Med 2006;30:474–9. 10.1016/j.amepre.2006.02.001 16704940

[42] Cruces G , Falcone G, Puig J. Differential price response for tobacco consumption: implications for tax incidence. Tob Control, 2022; 31: s95–100. 10.1136/tobaccocontrol-2021-056846 35017265

[43] Joseph RA , Chaloupka FJ. The influence of prices on youth tobacco use in India. Nicotine Tob Res 2014;16:S24–9. 10.1093/ntr/ntt041 23743096

[44] Farrelly MC , Bray JW, Pechacek T et al Response by adults to increases in cigarette prices by sociodemographic characteristics. South Econ J 2001;68:156–65. 10.2307/1061518

[45] Horn K , Fernandes A, Dino G et al Adolescent nicotine dependence and smoking cessation outcomes. Addict Behav 2003;28:769–76. 10.1016/S0306-4603(02)00229-0 12726789

[46] US National Cancer Institute and World Health Organization. The Economics of Tobacco and Tobacco Control (NIH Publication No. 16-CA-8029A). Bethesda, MD: National Cancer Institute, 2016.

[47] Verguet S , Kearns PKA, Rees VW. Questioning the regressivity of tobacco taxes: a distributional accounting impact model of increased tobacco taxation. Tob Control 2021;30:245–57. 10.1136/tobaccocontrol-2019-055315 32576701 PMC8077213

[48] Warner K. Smoking and health implications of a change in the federal cigarette excise tax. In: Isaacs SI, Knickman KR (eds.), Robert Wood Johnson Series on Health Policy: Tobacco Control Policy, 1st edn. San Francisco: Jossey Bass, 2006, 115–25.

[49] Aiken LS , West SG. Multiple Regression: Testing and Interpreting Interactions. Newbury Park, CA: Sage, 1991.

[50] *Millennials Increase Their C-Store Foodservice Visits*. Convenience Store News, 2019. https://csnews.com/millennials-increase-their-c-store-foodservice-visits (19 February 2026, date last accessed).

[51] Tobacco Industry Targeting Young People. Tobacco Control Research Group, University of Bath, 2025. https://www.tobaccotactics.org/article/tobacco-industry-targeting-young-people (19 February 2026, date last accessed).

